# Intestinal Parasitosis in Relation to CD4+T Cells Levels and Anemia among HAART Initiated and HAART Naive Pediatric HIV Patients in a Model ART Center in Addis Ababa, Ethiopia

**DOI:** 10.1371/journal.pone.0117715

**Published:** 2015-02-06

**Authors:** Hylemariam Mihiretie Mengist, Bineyam Taye, Aster Tsegaye

**Affiliations:** 1 Department of Medical Laboratory Sciences, Faculty of Medical and Health Sciences, Wollega University, Nekemte, Ethiopia; 2 Department of Medical Laboratory Sciences, School of Allied Health Science, College of Health Science, Addis Ababa University, Addis Ababa, Ethiopia; The University of Texas at San Antonio, UNITED STATES

## Abstract

**Background:**

Intestinal parasites (IPs) are major concerns in most developing countries where HIV/AIDS cases are concentrated and almost 80% of AIDS patients die of AIDS-related infections. In the absence of highly active antiretroviral therapy (HAART), HIV/AIDS patients in developing countries unfortunately continue to suffer from the consequences of opportunistic and other intestinal parasites. The aim of the study was to determine the prevalence of intestinal parasites in relation to CD4+ T cells levels and anemia among HAART initiated and HAART naïve pediatric HIV patients in a Model ART center in Addis Ababa, Ethiopia.

**Methods:**

A prospective comparative cross-sectional study was conducted among HAART initiated and HAART naive pediatric HIV/AIDS patients attending a model ART center at Zewditu Memorial Hospital between August 05, 2013 and November 25, 2013. A total of 180 (79 HAART initiated and 101 HAART naïve) children were included by using consecutive sampling. Stool specimen was collected and processed using direct wet mount, formol-ether concentration and modified Ziehl-Neelsen staining techniques. A structured questionnaire was used to collect data on socio-demographic and associated risk factors. CD4+ T cells and complete blood counts were performed using BD FACScalibur and Cell-Dyn 1800, respectively. The data was analyzed by SPSS version 16 software. Logistic regressions were applied to assess any association between explanatory factors and outcome variables. P values < 0.05 were taken as statistically significant.

**Results:**

The overall prevalence of IPs was 37.8% where 27.8% of HAART initiated and 45.5% of HAART naive pediatric HIV/AIDS patients were infected (p < 0.05). *Cryptosporidium* species, *E. histolytica/dispar*, *Hook worm* and *Taenia* species were IPs associated with CD4+ T cell counts <350 cells/μμL in HAART naive patients. The overall prevalence of anemia was 10% in HAART and 31.7% in non-HAART groups. *Hook worm*, *S. stercoralis* and *H. nana* were helminthes significantly associated with anemia in non-HAART patients [AOR, 95% CI: 4.5(1.3, 15.2), P< 0.05]. The prevalence of IPs in non-HAART patients was significantly associated with eating unwashed/raw fruit [AOR, 95%CI: 6.3(1.2, 25.6), P<0.05], open field defecation [AOR, 95%CI: 9.3(1.6, 53.6), P<0.05] and diarrhea [AOR, 95%CI: 5.2(1.3, 21.3), P<0.05]. IPs significantly increased in rural residents [AOR, 95%CI: 0.4(0.1, 0.9, P<0.05)].

**Conclusion:**

The overall prevalence of intestinal parasites significantly differed by HAART status and *cryptosporidium* species were found only in HAART naïve patients with low CD4+ T cell counts. Anemia was also more prevalent and significantly associated with IPs in non-HAART patients. This study identified some environmental and associated risk factors for intestinal parasitic infections. Therefore, Public health measures should continue to emphasize the importance of environmental and personal hygiene to protect HIV/AIDS patients from infections with intestinal parasites and maximize the benefits of HAART.

## Introduction

Intestinal parasitic infection (IPI) is a serious public health problem throughout the world particularly in developing countries [1–4]. In children, intestinal parasitic infections, particularly soil-transmitted helminthiases are the cause of common health problems in tropical countries. Several factors like climatic conditions, poor sanitation, unsafe drinking water, and lack of toilet facilities are the main contributors to the high prevalence of intestinal parasites in the tropical and sub-tropical countries. Furthermore, lack of awareness about mode of transmission of parasitic infections increases the risk of infection [2].

With impaired immunity especially in patients with CD4+ T cell counts < 200 cells/mm^3^, infections with opportunistic intestinal parasites result in diarrheal symptoms. With the introduction of HAART which partially restores the immune function, the incidence of opportunistic parasite infection such as cryptosporidiosis has declined [5–8]. Anemia, which can be mild, moderate or severe, is one of the several complications associated with intestinal parasitic infections. Although, anemia and intestinal parasitic infections have been reported as comorbidities in HIV infected patients [6], there is paucity of information in Ethiopia if these triple burdens (HIV, intestinal parasitic infections and anemia) coexist and associate with CD4+ T cell levels in HIV infected children.

In Ethiopia, intestinal parasitic diseases are among the ten top causes of morbidity nationwide. Most of the intestinal parasites are more common and their manifestations are more severe in children than adults. Infection in children is also associated with malnutrition, growth retardation and poor school performance [9]. Most of the previous comparative studies conducted in Ethiopia had focused on the prevalence and distribution of intestinal parasitic infections mainly in HAART taking and HAART naive adults but the pediatric population is usually unexplored.

## Materials and Methods

### Study setting and context

A prospective comparative cross-sectional study was conducted in Zewditu Memorial Hospital (ZMH), Addis Ababa, Ethiopia between August 05 and November 25, 2013. The hospital was selected because there are large numbers of pediatric HIV patients under follow up care in its ART clinic, which is a model center in the country. Pediatric HIV testing, counseling and ART are among the many services the hospital is providing. During data collection, there were more than 1250 pediatric HIV patients registered for follow up of which around 550 were on HAART and the remaining were at pre-HAART stage.

### Sample size and sampling technique

Since there are two populations (HAART initiated and HAART naive) and the study aims to compare intestinal parasitosis between the two populations, the sample size was calculated as follows;

$$let\;P=\frac{P1+rP2}{1+r}$$

$$n1=\frac{[Z\frac{a}{2}\sqrt{P\left(1-P\right)(1+\frac{1}{r}})+Zb\sqrt{\text{P}1\left(1-\text{P}1\right)\frac{P2\left(1-P2\right)}{r}}]2}{{\left(P1-P2\right)}^{2}}$$

[10]. Where;

P1 is the prevalence of parasites in HAART initiated patients

P2 is prevalence of parasites in HAART naive patients

P is the average prevalence proportion in the two groups (P = P1+rP2/1+r) = 0.65

According to a previous study, P1 is 0.50 and P2 is 0.77 [11]. With a precision of 5% and power of 80%, n1 is 72; n2 is *r*n1, where *r* is the ratio of N2 to N1 (700/550 = 1.3). Thus n2 is 94.

N1 is the number of HAART initiated pediatric HIV patients found in the hospital (550) and N2 is the number of HAART naive pediatric HIV patients found in the hospital (700) during data collection. So taking 10% contingency, n1 = 80 and n2 = 104 and a total of 184 study participants with 4 non respondents were enrolled in the study using consecutive sampling technique.

### Study population and data collection

Only pediatric HIV patients (age less than 18 years), who did not take anti-helminthes in the past two weeks, above 6 months of HAART duration (for HAART group only) and without any other acute/chronic disease causing immunosuppression and/or anemia, were enrolled. Parents or guardians were informed about the objective of the study. Then stool specimens were collected from each participant after getting written consent. Structured questionnaires were used to assess independent variables. Complete blood count and CD4+ T cell count, from EDTA whole blood using Cell-Dyn 1800 and FACScalibur, respectively, are routinely performed for patients on follow up visits. Thus CD4+ T cell count and hemoglobin level were measured simultaneously with stool specimen collection. Therefore, no blood specimen was collected for the purpose of this study. Standard operating procedures (SOPs) were followed during specimen collection and for all other laboratory procedures. All reagents used were checked for their expiry date and prepared according to the manufacturer’s instructions. Training was given for the data collectors to minimize biases.

### Specimen processing

Stool specimen collection and direct wet mount **analysis:** A single stool specimen was collected from each patient using clean, dry, leak proof and wide-mouthed caps and labeled. Stool specimen was obtained from all patients selected for the study. A direct saline and iodine wet mount of each sample were used to detect intestinal parasites microscopically. The wet mounts were examined under light microscope at 100X and 400X magnifications. A small portion of the stool specimen was also preserved in 10% formalin for repeating the tests whenever required for further analysis [12].

**Formol—Ether concentration method:** A portion of each preserved stool specimen was taken and processed following standard procedures. Briefly, 1 gm stool was placed in a clean conical centrifuge tube containing 7 mL 10% formol water by using applicator stick. The resulting suspension was filtered through a sieve into another conical tube. After adding 3–4 ml of diethyl ether to the formalin solution, the content was centrifuged at 3200 rpm for 1 minute. The supernatant was discarded; smear was prepared from the sediment and observed under light microscope with a magnification of 100X and 400X after air dried [13].

**Modified Ziehl Neelsen** (**Z-N) method:** A small portion of the concentrated stool specimen was processed for detection of opportunistic intestinal parasites using the Modified Ziehl Neelsen method. Thin smear was prepared directly from the sediment of concentrated stool and allowed to air dry. The slides were fixed with methanol for 1 minute and stained with carbol fuchsine for 30 minutes. After washing the slides in tap water, they were decolorized with 1% acid alcohol for 1–2 minutes and counterstained in 0.3% methylene blue for 60 seconds. Then the slides were washed in tap water, air dried and observed under light microscope with a magnification of 1000X [13]. The positive slides for oocysts were cross checked by experienced laboratory professionals in ZMH and the Ethiopian Public Health Institute (EPHI).

### Definition of anemia and immunosuppression

Anemia and immunosuppression were defined based on WHO 2011 and 2005 criteria, respectively [14, 15]. Thus, anemia was defined as mild when Hemoglobin (Hb) levels are between 10–10.9 g/dl for children aged under 5 and 11–11.9 g/dl for under 18 years; moderate when Hb is 7.0–9.9 g/dl for under 5 and 8.0–10.9 g/dl for under 18 years; severe when Hb <7.0 g/dl for under 5 and < 8.0 g/dl for under 18 years [14]. Immunosuppression states were defined as mild when CD4+T lymphocyte (CD4) counts are between 350–499 cells/μL for under 18 or between 25–35% for under 5 children, advanced when 200–349 cells/μL for under 18 or 15–25% for under 5 children and severe when CD4 count < 200 cells/ μL for under 18 or less than 15% for under 5 children) [15].

### Statistical analysis

The data were checked for errors, coded and double entered using EpiData version 3.1. SPSS version 16 (SPSS INC, Chicago, IL, USA) was used for data entry and analysis. Binary logistic regression was used to determine the association between intestinal parasites and demographic and clinical variables like diarrhea, regimen type, abdominal symptoms, HIV stage and lifestyle. Multiple logistic regressions were used to control for confounding factors. P values less than 0.05 were taken as statistically significant.

### Ethical considerations

The study was conducted after it was ethically reviewed and approved by the Research and Ethical Committee of the Department of Medical Laboratory Sciences, Addis Ababa University. Ethical clearance was also obtained from Addis Ababa Health Bureau. Then a letter informing the Hospital administrators was written from the Health Bureau and Permission obtained from Zewditu Memorial Hospital. All the information obtained from the study participants was coded to maintain confidentiality and data was collected after written informed consent was obtained. The positive results were timely reported to the clinicians for appropriate intervention.

## Results

### Characteristics of the study participants

A total of 180 (97.8% response rate) study participants, 79(43.9%) on HAART and 101(56.1%) HAART naive were consecutively enrolled. Male cases were predominant in both groups. The majority of HAART (97.5%) and non-HAART (95%) study participants were in the age range of 5–17 years with a respective median age of 12 years (range 4–17 years) and 11 years (range 0.3–17 years). Majority of HAART and non-HAART study participants 76 (96.2%) and 81 (80.2%) were urban residents, respectively. The assessment of educational status showed that 68(86%) of HAART and 83 (82%) of non-HAART participants were at primary school level. In binary logistic regression analysis; age group, educational status and gender did not show association with IPI in both groups (P>0.05), while participants of urban residents were more than twice less likely to harbor IPs regardless of HAART status [AOR, 95% CI: 0.4(0.1, 0.9) P< 0.05] (Table 1). Among patients on HAART, only 1(1.3%) was taking second line regimen whilst the remaining 78(98.7%) were on first line regimen. Most of them 45(57%) were on 4c (AZT+3TC+NVP) regimen followed by 4a (d4T+3TC+NVP) 20(25.3%), 4d (AZT+3TC+EFV) 10(12.7%), 4g (ABC+3TC+AZT) 2(2.5%), 4b (d4T+3TC+EFV) 1(1.3%) and 5a (ABC+DDI+LPV/r) 1 (1.3%).

**Table 1:** Associations of socio-demographic factors with prevalence of IPs by HAART status using binary and multiple logistic regressions in pediatric HIV/AIDS patients attending ZMH from August 5, 2013 to November 25, 2013, Addis Ababa, Ethiopia (N = 180).

Prevalence of intestinal parasites (IPs)									
Variables		HAART (n = 79)		Non-HAART (N = 101)		Total (N = 180)		COR(95%CI)	AOR(95% CI)
		Pos N(%)	Neg N(%)	Pos N(%)	Neg N(%)	Pos N(%)	Neg N(%)		
Age gy	< 2	0(0)	0(0)	1(33.3)	2(66.7)	1(33.3)	2(66.7)	1(1,1)	
	2–5	1(50)	1(50)	1(33.3)	2(66.7)	2(40)	3(60)	0.6(0.04,9.8)	
	6–11	9(28)	23(72)	19(36.5)	33(63.5)	28(33.3)	56(66.7)	1.2(0.2,6.8)	
	12–18	12(26.7)	33(73.3)	24(54.5)	19(45.5)	36(40.4)	52(59.6)	1	
Gender	Male	9(20.5)	35(79.5)	25(46.3)	29(53.7)	34(34.7)	64(65.3)	1.3(0.7,2.4)	
	Female	13(37)	22(63)	20(42.6)	27(57.4)	34(41.5)	48(58.5)	1	
Resid.	Urban	23(30)	54(70)	32(39.5)	49(60.5)	55(34.8)	103(65.2)	1	1
	Rural	0(0)	3(100)	13(68.4)	6(31.6)	13(59)	9(41)	2.7(1.1,6.7)*	0.4(0.1,0.9) *
Edu s.	NB	0(0)	1(100)	1(33.3)	2(66.7)	1(25)	3(75)	1	
	KG	3(42.8)	4(57.2)	1(20)	4(80)	4(33.3)	8(66.7)	3.5(0.3,48)	
	Primary	17(25)	51(75)	38(45.8)	45(54.2)	56(37)	95(63)	2.3(0.4,11)	
	Second.	2(66.7)	1(33.3)	5(50)	5(50)	7(53.8)	6(46.2)	1.9(0.6,6)	

* ^Significant at P value < 0.05, AOR = adjusted odds ratio, COR = crude odds ratio, KG = Kindergarten, NB = Not begin, Second = Secondary, Age gy = Age group in years, Resid. = Residence, Edu s. = Educational status, IPs = Intestinal Parasites^

### Prevalence of IPs among HAART and non-HAART groups

**Fig 1:**
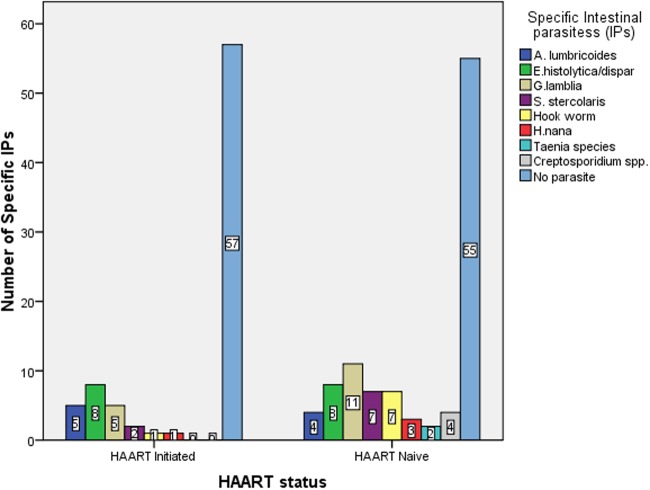
Distribution of species specific intestinal parasites among HAART initiated and HAART naïve pediatric HIV patients in ZMH from August 05, 2013 to November 25, 2013, Addis Ababa, Ethiopia.

From all the 180 pediatric HIV/AIDS patients participated in this study, intestinal parasites were detected in 68(37.8%) of them. Among the 79 pediatric HIV patients taking HAART, 22(27.8%) were infected with intestinal parasites. There were no opportunistic intestinal parasites detected in this group. The highest prevalent IP was *E*. *histolytica/dispar* accounting for 10% followed by *G*. *lamblia* 7.6%, *A*. *lumbricoides* 6.3%, *S*. *stercoralis* 2.5%, *Hook worm* and *H*. *nana* 1.3% each (Fig. 1).

The overall prevalence of IPs in the non-HAART arm was 46(45.5%) and 4(3.96%) were infected with *Cryptosporidium* species. The most prevalent IP was *G*. *lamblia* (11%) followed by *E*. *histolytica/dispar*, *Hookworm*, and *S*. *stercoralis* (6.9% each), *Ascaris lumbricoides* (4%), *Cryptosporidium species* (3.96%), *Taenia species* (3%) and *H*. *nana* (2%) (Fig. 1). There was no statistically significant difference in the prevalence of species specific IPs in both groups in binary logistic regression analysis (P>0.05). But the overall prevalence of IPs was higher in non-HAART (2.2 times more likely to be infected with IPs) which was 45.5% compared to HAART (27.8%) indicating statistically significant decrease of IPs partly as a result of HAART use [OR, 95%CI: 2.2(1.2, 4.0), P<0.05] (Table 2).

**Table 2:** Relationship between HAART status and infection with IPs among pediatric HIV/AIDS patients in ZMH from August 5, 2013 to November 25, 2013, Addis Ababa, Ethiopia (N = 180).

Variable		Infection with IPs		OR(95% CI)	P-value
		Yes N(%)	No N(%)		
HAART S	On HAART (n = 79)	22(27.8%)	57(72.2%)	1	
	Non-HAART(n = 101)	46(45.5%)	55(54.5%)	2.2(1.2,4.0)	0.016*
HAART D	6–24 months	15(65.2)	8(34.8)	3.3(0.33,11.2)	0.31
	24–36 months	9(31)	29(69)	1.2(0.67,5.32)	0.43
	>36 months	2(11)	16(89)	1	

* ^Significant at P value< 0.05, OR = Odds ratio, IPs = Intestinal Parasites, HAART S = HAART status, HAART D = HAART Duration^

### Association of IPs and CD4+ T cell counts

Of the 79 study participants who were on HAART, 66 patients (83.5%) had CD4 count > 350 cells/μL and the remaining 13 patients (16.5%) had CD4 count < 350 cells/μL. Among the 13 HAART patients with CD4 count < 350 cells /μL, parasites were identified in 4 (30.8%) of them while 27.3% of those with CD4 count>350 had IPs, showing no statistically significant association with CD4+ T cell levels [AOR, 95% CI: 1.2(0.3, 4.3), P = 0.8) (Table 3).

**Table 3:** The effect of CD4+ T cell count on IPs among pediatric HIV/AIDS patients in ZMH from August 5, 2013 to November, 2013, Addis Ababa, Ethiopia (N = 180).

Variables			Intestinal Parasites		AOR(95%CI)	P
			Present N(%)	Absent N(%)		
HAART	CD4+ T cell	<350	4(30.8)	9(69.2)	1.2(0.3,4.3)	0.8
		≥350	18(27.3)	48(72.7)	1	
N-HAART	CD4+ T cell	<350	28(80)	7(20)	7.3(1.5,35)	0.013*
		≥350	17(25.8)	49(74.2)	1	

* ^Significant at P< 0.05, AOR = Adjusted odds ratio, CD4+ T cell = CD4+ T cell count (cells/mm3), P = P-value, N-HAART = Non-HAART^

From all the study participants who were not on HAART, 66 patients (65.3%) had CD4 count > 350 cells/μL while 35 patients (34.7%) had a count < 350 cells/μL. Among the 35 non-HAART patients with CD4 count < 350 cells /μL, parasites were identified in 28 (80%) patients which was much higher compared to those with CD4+ T cells levels above 350 cells/μL which was 25.8%. Those having CD4+ T cell counts below 350 cells /μL were about 7.3 times more likely to harbor intestinal parasites than those having CD4+ T cell cell counts above 350 cells/μL [AOR, 95%CI: 7.3(1.5, 35), P< 0.05) (Table 3).

Among all HAART initiated pediatric HIV patients, there was no specific parasite identified to be significantly associated with CD4 levels < 350 cells/μL in binary logistic regression analysis (Table 4). But in case of HAART naive pediatric HIV patients, *E*. *histolytica/dispar* [AOR, 95%CI: 9.33(1.7, 50.7), P<0.05], *Hook worm* [AOR, 95%CI: 9(1.7, 50), P<0.05], *Taenia species* [AOR, 95%CI: 14(1.1, 175), P<0.05] and *Cryptosporidium species* [AOR, 95%CI: 13(10.5, 97.6), P<0.01] were significantly associated with CD4 levels < 350 cells/μL (Table 5).

**Table 4:** Binary logistic regression analysis of specific IPs in relation to CD4+ T cell levels among HAART initiated pediatric HIV patients in ZMH from August 5, 2013 to November 25, 2013, Addis Ababa, Ethiopia (N = 79).

Specific parasites			COR	95% CI	P- value
*A.lumbricoides*	CD4+ T cell	< 350	0.00	1,1	0.1
		≥350	1		
*E. histolytica/dispar*	CD4+ T cell	< 350	0.8	0.1,7.9	0.9
		≥350	1		
*G. lamblia*	CD4+ T cell	< 350	2.73	0.5,19	0.23
		≥350	1		
*S. stercoralis*	CD4+ T cell	< 350	1.0	0, 1	1.0
		≥350	1		
*Hook worm*	CD4+ T cell	< 350	1.0	0,1	1.0
		≥350	1		
*H. nana*	CD4+ T cell	< 350	1.0	0,1	1.0
		≥350	1		

^COR: crude odds ratio, CD4+ T cell = CD4+ T cell count (cells/mm3)^

**Table 5:** Relationship between specific IPs and CD4+ T cell levels among HAART naive pediatric HIV patients in ZMH from August 5, 2013 to November 25, 2013, Addis Ababa, Ethiopia (N = 101).

Specific parasites			COR(95% CI)	P	AOR(95%CI)	P
*A.lumbricoides*	CD4+ T cell	< 350	1.5(0.2,4.3)	0.67		
		≥350	1			
*E. histolytica/dispar*	CD4+ T cell	< 350	3.8(1.8,9.2)	0.046*	9.33(1.7,50.7)	0.010*
		≥350	1		1	
*G. lamblia*	CD4+ T cell	< 350	0.5(0.1,0.9)	0.034*	2.73(1,17)	0.06
		≥350	1		1	
*S. stercoralis*	CD4+ T cell	< 350	1.2(0.7,4.3)			
		≥350	1			
*Hook worm*	CD4+ T cell	< 350	7.9(1.1,12)		9.33(1.7,50.7)	0.010*
		≥350	1		1	
*H. nana*	CD4+ T cell	< 350	7(0.3,167)			
		≥350	1			
*Taenia species*	CD4+ T cell	< 350	3.2(1.4,7.6)	0.044*	14.0(1.1,75.5)	0.041*
		≥350	1		1	
*Cryptosporidium*	CD4+ T cell	< 350	8.9(1.6,33)		13.2(10.5,76.2)	0.0013**
		≥350	1		1	

^AOR = adjusted odds ratio,^

*^significant at P value <0.05,^

**^highly significant at P<0.01, COR = Crude odds ratio, CD4+ T cell = CD4+ T cell count (cells/mm3), P = P-value^

### Association of IPs and anemia

From the total of 79 HAART initiated pediatric HIV patients, 8(10%) were anemic (mean hemoglobin level 13.36±1.35g/dl) of which 4(50%) were infected with IPs. Most of anemic patients used 4c (AZT+3TC+NVP) 4(44.4%) and 4d (AZT+3TC+EFV) 2(22.3%) drug regimens. But none of the regimens showed significant association with IPIs.

Unlike those on HAART, 32(31.7%) of HAART naive pediatric HIV patients were anemic (mean hemoglobin level 12.89±1.9 g/dl) of which 24(75%) were infected with IPs. Whereas the presence of intestinal parasites was not significantly associated with anemia in HAART initiated patients (COR, 95% CI = 2.9(0.7, 13), P = 0.15), in the non HAART group anemic patients had 4.5 times likelihood of being infected with intestinal parasites compared to non-anemic patients [AOR, 95% CI: 4.5(1.3, 15.2), P< 0.05] (Table 6). In non-HAART patients, *Hookworm*, *S*. *stercoralis* and *H*. *nana* were significantly associated with anemia (Table 7).

**Table 6:** Relationship between anemia and IPs among pediatric HIV/AIDS patients in ZMH from August 5, 2013 to November 25, 2013, Addis Ababa, Ethiopia (N = 180).

Variables			Intestinal Parasites		AOR (95%CI)	P
			Present N(%)	Absent N(%)		
HAART	Anemia	Present	4(50)	4(50)	1.2(0.5,3.4)	0.87
		Absent	18(25.4)	53(74.6)	1	
Non- HAART	Anemia	Present	24(75)	8(25)	4.5(1.3,15.2)	0.016*
		Absent	21(30.4)	48(69.6)	1	

* ^Significant at P< 0.05, AOR = Adjusted odds ratio, IPs = Intestinal parasites, P = P-value^

**Table 7:** Association between specific IPs and status of anemia (mild, moderate, sever) among HAART naive pediatric HIV patients in ZMH from August 5, 2013 to November 25, 2013, Addis Ababa, Ethiopia (N = 101).

Specific parasites	Anemia status					
	Mild N(%)	P	Moderate N(%)	P	Sever N(%)	P
*A.lumbricoides*	0(0)	0.6	0(0)	0.8	0(0)	0.98
*E. histolytica/dispar*	2(66.7)	0.2	1(33.3)	0.18	0(0)	0.96
*G. lamblia*	3(75)	0.17	1(25)	0.34	0(0)	0.95
*S. stercoralis*	1(14.3)	0.08	6(85.7)	0.003**	0(0)	0.90
*Hook worm*	4(57)	0.0026**	2(28.6)	1	1(14.4)	0.99
*H. nana*	0(0)	0.83	1(100)	0.003**	0(0)	0.98
*Taenia species*	1(100)	0.33	0(0)	1	0(0)	0.99
*Cryptosporidium*	0(0)	0.67	1(100)	0.13	0(0)	0.99

^AOR: adjusted odds ratio,^

**^highly significant at P<0.01, P = P-value^

**Fig 2:**
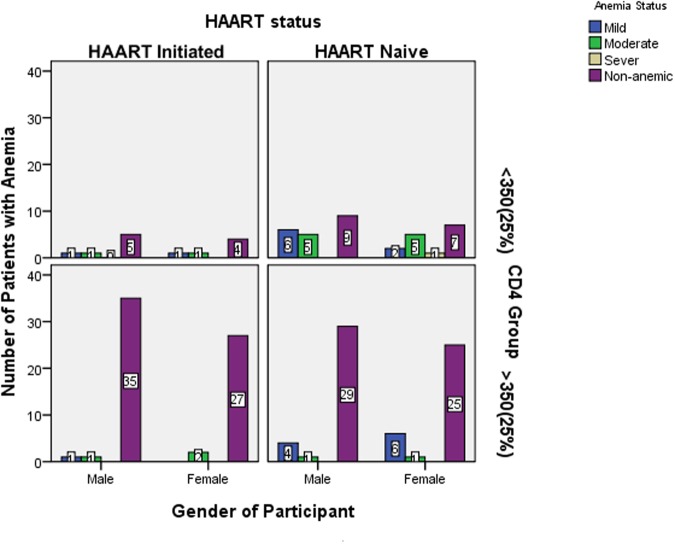
Distribution of anemia status/category with HAART status, CD4+ T cell category and gender among HAART initiated and HAART naïve pediatric HIV patients in ZMH from August 05, 2013 to November 25, 2013, Addis Ababa, Ethiopia.

Even though distribution of anemia status was higher in females with lower CD4 +T cell counts in HAART naïve groups, it was not statistically significant regardless of HAART in relation to gender (Fig. 2).

### Prevalence of IPs with associated factors

Table 8 shows that among the 79 pediatric patients on HAART, 17(21.5%) were diarrheic of which 8(47%) were infected with intestinal parasites. Comparatively, the number of diarrheic patients in the non-HAART groups was high, 35.6% (36/101). In binary logistic regression; presence of toilet, hand washing habit, playing with soil, eating raw meat, wearing closed shoe, source of drinking water and finger nail trim were not significantly associated with IPI (P>0.05). On the other hand, the prevalence of intestinal parasites in non-HAART group was significantly associated with eating unwashed/raw fruit [AOR, 95%CI: 6.3(1.2, 25.6), P<0.05], open field defecation [AOR, 95%CI: 9.3(1.6, 53.6), P<0.05] and diarrhea [AOR, 95%CI: 5.2(1.3, 21.3), P<0.05]. So those non-HAART patients who were diarrheic, ate unwashed/raw fruit, and defecated openly had 5.2, 6.3, and 9.3 times more likelihood of getting infection with intestinal parasites, respectively.

**Table 8:** Risk factors associated with IPs among HAART and HAART naive pediatric HIV patients with prevalence of intestinal parasites (N = 180) in ZMH from August 5, 2013 to November 25, 2013, Addis Ababa, Ethiopia.

Variables		Presence of parasite				COR 95% CI	P value	AOR95% CI	P value
		HAART Status							
		On HAART (n = 79)		Non-HAART (n = 101)					
		Pos N(%)	Neg N(%)	Pos N(%)	Neg N(%)				
Hand WAM	Yes	18(25)	54(75)	42(44.2)	53(55.8)	1			
	No	4(57)	3(43)	1(50)	1(50)	1.3(0.1,20)	0.87		
Eating URF	Yes	12(22.6)	41(77.4)	36(50.7)	35(49.3)	0.4(0.1,0.9)	0.04*	6.3(1.2,31)	0.023*
	No	10(38.5)	16(61.5)	7(27)	19(73)	1		1	
Eating RM	Yes	8(29.6)	19(70.4)	14(53.8)	12(46.2)	0.6(0.2,1.5)	0.26		
	No	14(28)	36(72)	29(40.8)	42(59.2)	1			
Open FD	Yes	21(29)	51(71)	39(55.7)	31(44.3)	0.2(0.1,0.4)	0.001**	9.3(1.6,53.6)	0.012*
	No	1(14.3)	6(85.7)	4(14.8)	23(85.2)	1		1	
Hand WAT	Yes	19(26.8)	52(73.2)	38(42.7)	51(57.3)	1			
	No	3(37.5)	5(62.5)	5(62.5)	3(37.5)	2.2(0.5,10)	0.3		
Finger NT	Yes	16(25.8)	46(74.2)	26(41.3)	37(58.7)	1			
	No	6(35.3)	11(64.7)	17(50)	17(50)	1.4(0.6,3.3)	0.4		
Wearing CS	Always	20(27)	54(73)	31(38.3)	50(61.7)	1		1	
	S/times	2(40)	3(60)	12(70.6)	5(29.4)	3.8(1.2,12)	0.019*	0.8(0.1, 4.5)	0.8
Playing WS	Yes	14(32.6)	29(67.4)	25(46.3)	29(53.7)	0.8(0.4,1.8)	0.6		
	No	8(22.2)	28(77.8)	18(41)	26(59)	1			
Diarrhea	Present	8(47)	9(53)	29(80.6)	7(19.4)	0.1(0.03,0.2)	0.000**	5.2(1.3,21.3)	0.022*
	Absent	14(22.6)	48(77.4)	16(24.6)	49(75.4)	1		1	
WHO stage	I	4(30.8)	9(69.2)	5(27.7)	17(72.3)	1		1	
	II	17(28.8)	42(71.2)	28(43.8)	36(56.2)	13.6(2.7,68.1)	0.001*	0.2(0.02,1.3)	0.09
	III	1(14.3)	6(85.7)	12(80)	3(20)	5.1(1.3,20)	0.018*	0.4(0.03,6.4)	0.56
	IV	0(0)	2(100)	0(0)	0(0)				

^AOR = adjusted odds ratio,^

*^significant at P value <0.05,^

**^highly significant at P<0.01, COR = crude odds ratio, Hand WAM = Hand washing after meal, Eating URF = Eating unwashed/raw fruit, Eating RM = Eating raw meat, Open FD = Open field defecation, Hand WAT = Hand washing after toilet, Finger NT = Finger nail trim, Wearing CS = Wearing closed shoe, Playing WS = Playing with soil, S/times = Some times^

## Discussion

The significantly higher prevalence of common and opportunistic intestinal parasites observed in the HAART naïve children in this study (45.5% versus 27.8%) partly signifies the value of HAART in reducing intestinal parasites through improving the immune response as also demonstrated by the lower prevalence in those with higher CD4+ T cell counts. Moreover, the observed 45.5% prevalence of intestinal parasite among HAART naive patients in this study is in line with reports from South west Ethiopia (44.8%) and Gondar (43.5%) [8, 21]. However, the finding is lower compared to studies of Indonesia (77%), Brazil (63.9%) and from selected ART centers of Adama, Afar and Dire-Dawa (52%) [11, 19, 23]. This low prevalence in this study might be due to geographic difference in sampling (more than one study area for most reports) and time gap where those studies were done some years ago. But nowadays there is a better awareness of the patients about intestinal parasite infection and their cause.

On the other hand, the 27.8% prevalence of IPs detected among patients on HAART in this study is higher than that reported in Brazil (24%) and Dessie (17.6%) [19, 24] but lower than from studies done in Indonesia (50%), in selected ART centers of Adama, Afar and Dire-Dawa (48%), Jimma (39.6%) and Addis Ababa (34.3%), [11, 23, 25, 26]. This lower prevalence might be due to better laboratory test supported follow-up and better awareness of the patients themselves in adopting prevention and treatment measures against intestinal parasites in our study area plus the combined effect of anti-helminthes and HAART may better decrease occurrence of IPs.

*Strogyloides stercoralis* is an important human parasite primarily because of its potential for serious and even lethal disease in immunosuppressed patients. The prevalence of *Strongyloides stercoralis* is higher in non-HAART (6.9%) than patients on HAART (2.5%) in the present study which is comparable with a study done in Nigeria (6.39%) [6]. But higher than studies done in Malaysia (0%), China (0%) and Dessie (0.7%) [17, 18, 24]. The possible reasons might be due to geographical differences, comparatively small sample size in this study and age differences i.e. pediatric patients have more exposure to soil than adults since they play in the field barefooted or wear open shoes. Moreover, differences in socioeconomic status could partly explain the observed variation with studies from Malaysia [17] and China [18].

The prevalence of *cryptosporidium species* (3.96%) in non-HAART patients in this study is much lower than the prevalence reported in Brazil (8.1%), Gondar (8.7%), Nekemte (25.5%), and selected ART centers of Adama, Afar and Dire-Dawa (8%) and Jimma (13.3%) [19, 21, 22, 23, 25] but higher compared to the study done in Dessie (1.5%) [24]. The existence of such variation may be explained by the difference in geographic location, better general hygiene of the population in Addis Ababa than the other regions of Ethiopia, and better follow up care. Besides, study participants were recruited without considering their diarrhea status in our study.

In case of non-HAART Pediatric HIV patients, *E*. *histolytica/dispar* [AOR, 95%CI: 9.33(1, 50), P<0.05], *Hookworm* [AOR, 95%CI: 9(1.7, 50), P<0.05], and *Taenia species* [AOR, 95%CI: 14(1, 175), P<0.05] were non opportunistic IPs significantly associated with CD4+ T cell levels < 350 cells/μL in this study. This result agrees with a study done in Malaysia [17] and Dessie (for only *E*. *histolytica/dispar*) [24].

Infection with *cryptosporidium species* and other intestinal parasites among non-HAART patients, as is documented by other studies in Ethiopia and Nigeria [20, 22, 23, 24], were significantly associated with having CD4 count <350 cells/μL. Whereas, no specific association was demonstrated in the HAART group. This may be due to the fact that opportunistic parasites are known to resolve spontaneously with immune restoration among HIV/AIDS patients on HAART [5].

The 31.7% prevalence of anemia in HAART naive patients in this study is lower than the findings from Nigeria (93.3%) [6]. This might be due to differences in age groups (factors other than HIV and intestinal parasites also contribute to anemia in children). *Hookworm*, *S*. *stercoralis*, and *H*. *nana* were associated with anemia which is in line with a study done in Veitnam [16] where *Hookworm* and *Trichuris trichuira* were associated with anemia, but different (except for *Hookworm*) from a study done in Nigeria where *Cryptosporidiasis*, *Ascariasis*, *Hook worm* and *Taeniasis* were associated with anemia [6]. These differences might be due to low prevalence of *Cryptosporidiasis*, *Taeniasis* and *Ascariasis* in this study.

In the present study, only 8 (10%) of HAART initiated pediatric patients were anemic from which 4(50%) are infected with IPs. However, the presence of intestinal parasites was not significantly associated with anemia in HAART initiated patients which is different from a study done in Nigerian adults where the presence of IPs was associated with anemia [6]. In addition to age, difference in the duration of HAART where inclusion of those with 3–6 months of HAART initiation in Nigerian case might explain the difference with our study.

The total prevalence of diarrhea detected in the present study (29.4%) was consistent with a study done in Nekemte (27%) [22], but lower than a study from Southwest Ethiopia (51.1%) and selected ART centers of Adama, Afar and Dire-Dawa (37.5%) [8, 23]. The possible explanation might be due to better life style in urban settings (like Addis Ababa), appropriate use of anthelminthic and partly could be due to low prevalence of opportunistic intestinal parasites.

Non-HAART initiated patients who were diarrheic, ate unwashed/raw fruit, and defecate openly had 5.2, 6.3, and 9.3 times likelihood of getting infection with intestinal parasites, respectively. Although the majority of our study participants reported that they have toilet and use pipe water for drinking, this result is almost similar to a study done in Dessie where absence of toilet, source of water and living condition were significantly associated with the presence of IPs [24].

In this study, we have used normal saline, iodine, formol-ether concentration and modified Ziehl-Neelsen staining methods for detection of common and opportunistic intestinal parasites. We have not used water-ether sedimentation method for Microsporidia and other sensitive methods, which might result in underestimation of the prevalence of intestinal parasites.

## Conclusion

The prevalence of intestinal parasites was found to be higher in HAART naïve than HAART experienced patients. High proportions of intestinal parasites were associated with lower CD4+ T cell counts in HAART naïve initiated patients. Infections with opportunistic intestinal parasites were associated with lower CD4+ T cell counts in HAART naïve patients only. Increasing the immune status of HIV infected patients with antiretroviral therapy may help to reduce acquisition of parasites. Moreover, the prevalence of anemia was higher in HAART naïve patients where helminthes like *Hookworm*, *Strogyloides stercoralis* and *Hymenolopis nana* were IPs associated with anemia. Open field defecation, eating raw/unwashed fruit, diarrhea and being HAART naïve significantly increased the prevalence of intestinal parasites in the study area. Public health measures, parasitological tests and large scale longitudinal studies to understand casual relationships are recommended in the study setting.
